# Impact of Multiplex Polymerase Chain Reaction Test in Patients With Meningitis or Encephalitis

**DOI:** 10.1093/ofid/ofad634

**Published:** 2023-12-18

**Authors:** Daisuke Kitagawa, Taito Kitano, Yuto Uchihara, Takafusa Ando, Hiroki Nishikawa, Rika Suzuki, Masayuki Onaka, Takehito Kasamatsu, Naoyuki Shiraishi, Kiyoshi Takemoto, Madoka Sekine, Soma Suzuki, Yuki Suzuki, Akiyo Nakano, Ryuichi Nakano, Hisakazu Yano, Sayaka Yoshida, Makoto Kawahara, Koichi Maeda, Fumihiko Nakamura

**Affiliations:** Department of Laboratory Medicine, Nara Prefecture General Medical Center, Nara, Japan; Department of Microbiology and Infectious Diseases, Nara Medical University, Kashihara, Japan; Johns Hopkins Bloomberg School of Public Health, Baltimore, USA; Department of Neurology, Nara Prefecture General Medical Center, Nara, Japan; Department of Neurology, Nara Prefecture General Medical Center, Nara, Japan; Department of Pediatrics, Nara Prefecture General Medical Center, Nara, Japan; Department of Pediatrics, Nara Prefecture General Medical Center, Nara, Japan; Department of Pediatrics, Nara Prefecture General Medical Center, Nara, Japan; Department of Infectious Diseases, Nara Prefecture General Medical Center, Nara, Japan; Department of Infectious Diseases, Nara Prefecture General Medical Center, Nara, Japan; Department of Critical Care Medicine, Nara Prefecture General Medical Center, Nara, Japan; Department of Laboratory Medicine, Nara Prefecture General Medical Center, Nara, Japan; Department of Laboratory Medicine, Nara Prefecture General Medical Center, Nara, Japan; Department of Microbiology and Infectious Diseases, Nara Medical University, Kashihara, Japan; Department of Microbiology and Infectious Diseases, Nara Medical University, Kashihara, Japan; Department of Microbiology and Infectious Diseases, Nara Medical University, Kashihara, Japan; Department of Microbiology and Infectious Diseases, Nara Medical University, Kashihara, Japan; Department of Pediatrics, Nara Prefecture General Medical Center, Nara, Japan; Department of Neurology, Nara Prefecture General Medical Center, Nara, Japan; Department of Infectious Diseases, Nara Prefecture General Medical Center, Nara, Japan; Department of Laboratory Medicine, Nara Prefecture General Medical Center, Nara, Japan

**Keywords:** antibacterial, antiviral, days of therapy, FilmArray, meningitis

## Abstract

**Background:**

The objective of this study was to evaluate the impact of the FilmArray meningitis/encephalitis panel (FAME) on length of stay (LOS) and duration of antimicrobial treatment in children and adults in a Japanese community hospital.

**Methods:**

This retrospective cohort study was conducted in Japan between January 2016 and December 2022. We included hospitalized patients with cerebrospinal fluid (CSF) samples and those aged <2 months or who had 5 or more white blood cells/μL in the CSF. To compare the days of therapy (DOT) and LOS between the pre-FAME and FAME periods, multivariate Poisson regression analyses were conducted without an offset term.

**Results:**

The number of cases undergoing pathogen-specific polymerase chain reaction increased from 3.7% in the pre-FAME period to 57.5% in the FAME period (*P* < .001). The pathogen identification rate also increased during the FAME period, from 0.4% to 18.7% (*P* < .001). While the antibacterial DOT was not statistically different between the 2 periods (adjusted rate ratio [aRR], 1.06 [95% confidence interval {CI}, 1.00–1.13]; *P* = .063]), the antiviral DOT was significantly shorter in the FAME period (aRR, 0.80 [95% CI, .71–.89]; *P* < .001).

**Conclusions:**

This study revealed a significant reduction in antiviral use during the FAME period, whereas LOS and antibacterial use did not decrease. Given the possibility of factors (eg, the COVID-19 pandemic) affecting the epidemiology of meningitis and encephalitis, the indications and impact of the FAME test should be evaluated with continuous monitoring of the epidemiology of meningitis and encephalitis and its clinical impact.

Meningitis is an important cause of morbidity and mortality worldwide [1]. The rapid identification of causative organisms and timely initiation of appropriate treatment are crucial to improve the outcome of meningitis. However, conventional bacterial culture methods can identify only a fraction of the causal organisms of bacterial meningitis, and it typically takes 1 or 2 days to obtain the result [2–6]. While molecular diagnoses, including polymerase chain reaction (PCR), have been used to identify organisms, hospitals without in-house molecular laboratories are unable to conduct molecular tests rapidly [2–6]. Without rapid identification of causative organisms in cases with suspected meningitis, the duration of antimicrobial treatment may be prolonged.

The FilmArray meningitis/encephalitis panel (FAME) is a multiplex PCR assay that detects 14 types of causal organisms of meningitis within 80 minutes. The sensitivity and specificity of FAME have been evaluated [7–9], and the clinical impacts of FAME have also been evaluated in some US and European studies [10–13]. A pediatric study from the United States revealed shorter durations of intravenous antimicrobial therapy and more frequent diagnosis of infections after the introduction of FAME, but there was no difference in the hospital length of stay (LOS) [10]. A study among adults demonstrated that there was a significant decrease in the percentage of patients who received acyclovir, vancomycin, and ampicillin, but not other antimicrobials, or in the LOS, after implementation of the use of FAME [12].

The Japanese government approved the use of the FAME panel in 2019. While there is a pediatric study evaluating the impact of FAME on the duration of acyclovir treatment, there is little evidence regarding the overall clinical impact of FAME among Japanese patients [14]. The objective of this study was to evaluate the impact of FAME on LOS and duration of antimicrobial treatment among both children and adults in a Japanese community hospital.

## METHODS

### Study Design and Settings

This retrospective cohort study was conducted at the Nara Prefecture General Medical Center in Japan. The study period was between January 2016 and December 2022. As FAME was introduced at our center in December 2020, the pre-FAME period was from January 2016 to November 2020 and the FAME period was from December 2020 to December 2022.

During the pre-FAME period, bacterial cultures were performed on cerebrospinal fluid (CSF) samples from patients with suspected meningitis. For some selected patients (ie, patients with suspected viral meningitis by specific pathogens such as herpes simplex virus (HSV) and varicella zoster virus (VZV), viral PCR tests, including PCR tests for HSV-1/2, VZV, parvovirus, human herpesvirus 6 (HHV-6), and enterovirus were conducted at external laboratories within 2–4 days of the typical turnaround time. During the FAME period, CSF samples from patients with suspected meningitis were tested for identification of bacteria by culture and, if required, by FAME. FAME was conducted during the daytime (8:30 Am–5:15 Pm) on weekdays by trained laboratory staff.

Inclusion criteria were hospitalized patients with CSF samples and 1 of the following: patients aged <2 months or patients (irrespective of their age) who had ≥5 white blood cells/μL in the CSF. Patients from the neurosurgery and hematology/oncology departments were excluded. Immunocompromised patients and patients with neurosurgical devices were not excluded but were documented and presented in Table 1. The follow-up CSF samples were excluded from the study. While all results of the CSF tests were reported through electronic medical records, laboratory staff communicated directly with clinicians in case of positive results.

**Table 1.** Patient Characteristics and Days of Therapy During the Pre–FilmArray Meningitis/Encephalitis Panel (FAME) Period and FAME Period

Characteristic	Pre-FAME Period	FAME Period	*P* Value
	Jan 2016 to Nov 2020 (n = 519)	Dec 2020 to Dec 2022 (n = 193)	
Sex, female	230 (44.3%)	93 (48.2%)	.356
Age			
<2 mo	81 (15.6%)	26 (13.5%)	.004
2 mo–14 y	98 (18.9%)	16 (8.3%)	
15–59 y	155 (29.9%)	69 (35.7%)	
≥60 y	185 (35.6%)	82 (42.5%)	
CSF WBC ≥5 cells/μL	509 (98.1%)	185 (95.9%)	.094
CSF indices, median (10th–90th percentiles)			
CSF WBC count, cells/μL	18 (5–449)	27 (6–334)	.345
Multinuclear	3 (0–119)	2 (0–141)	.328
Mononuclear	12 (4–239)	20 (4–214)	.156
CSF RBC count, cells/μL	7 (0–6554)	11 (0–30 000)	
CSF protein, mg/dL	48 (23–153)	65 (30–239)	<.001
CSF glucose, mg/dL	61 (44–92)	59 (40–89)	.061
CSF chloride, mEq/L	120.3 (112.6–126.0)	120.4 (113.1–127.3)	.513
Hospitalization rate	474 (91.3%)	170 (88.1%)	.192
ICU admission rate	53 (10.2%)	25 (13.0%)	.298
Length of stay, d, median (10th–90th percentiles)	13 (4–49)	17 (5–55)	<.001
DOT, median (10th–90th percentiles)			
Total days of antimicrobial therapy	6.6 (0–25.4)	5.3 (0–25.8)	.034
Days of antibacterial therapy	4.5 (0–17.9)	2.0 (0–24.2)	.063
Days of antiviral therapy	0 (0–10.5)	0 (0–9.3)	<.001
Neurosurgical device	3 (0.6%)	2 (1.0%)	.515
Immunocompromising condition	17 (3.3%)	6 (3.1%)	.911

Data are presented as No. (%) unless otherwise indicated. Immunocompromising conditions included hematological malignancies, active treatment with noncorticosteroid immunosuppressive or immunomodulatory therapy for the past 12 months, and high-dose corticosteroid therapy (eg, ≥20 mg prednisone or equivalent/day for ≥2 weeks) for the past month.

Abbreviations: CSF, cerebrospinal fluid; DOT, days of therapy; FAME, FilmArray meningitis/encephalitis panel; ICU, intensive care unit; RBC, red blood cell; WBC, white blood cell.

### Patient Consent Statement

The data regarding antimicrobial prescriptions were obtained from the electronic medical records of the Nara Prefecture General Medical Center database, and the number of monthly antimicrobial prescriptions was calculated. The need for informed consent was waived by the medical ethics committee of the center owing to the retrospective nature of the study. The medical ethics committee of Nara Prefectural General Medical Center approved the experimental protocol (number 708). All experiments were performed in accordance with the relevant guidelines and regulations.

### FilmArray System

During the FAME period, physicians decided on which patients with suspected meningitis or encephalitis the FAME test should be conducted. The multiplex PCR was performed according to the manufacturer's instructions [15]. All specimens were processed individually in a biosafety cabinet, and appropriate personal protective devices such as masks, gloves, and gowns were worn during specimen processing. The FAME test automatically performs nucleic acid extraction, reverse transcription, nucleic acid amplification, and result analysis in a single assessment (1 sample) in approximately 80 minutes. The FilmArray system automatically analyzes and displays the results. If each target, “detected” or “not detected” result verifies the test result. If all panel targets display the result “invalid,” this indicates that one of the internal controls is inadequate. The pathogens detected by FAME among our patients were *Escherichia coli* K1, *Haemophilus influenzae*, *Listeria monocytogenes*, *Neisseria meningitidis*, *Streptococcus agalactiae*, *Streptococcus pneumoniae*, cytomegalovirus (CMV), enterovirus, HSV-1, HSV-2, HHV-6, human parechovirus, VZV, and *Cryptococcus* (*C neoformans/C gattii*).

### Outcome

The primary outcomes of this study were the total days of therapy (DOT) for any antimicrobials, including antibacterial and antiviral agents, and the LOS. The secondary outcomes were DOT for antibacterials and antivirals.

### Statistical Analysis

The characteristics of the study participants in the pre-FAME and FAME periods were compared using χ^2^ tests for categorical variables and nonparametric tests for continuous variables. To compare DOT and LOS between the pre-FAME and FAME periods, multivariable Poisson regression analyses were conducted without an offset term. The dependent variables were patient age (<2 months, 2 months–14 years, 15 years–59 years, and ≥60 years), sex, and seasonality (March–May, June–August, September–November, and December–February). Statistical analyses were performed using Stata version 14.2 software (College Station, Texas).

## RESULTS

A total of 712 patients were included in the study (519 in the pre-FAME period and 193 in the FAME period). Patient characteristics, CSF results, and clinical data are presented in Table 1.

CSF examination showed no significant differences between the pre-FAME and FAME periods, except for CSF protein levels (median of 48 and 65 mg/dLin the pre-FAME and FAME periods, respectively; *P* < .001; Table 1).

Microbiological testing demonstrated that the number of cases undergoing pathogen-specific PCR increased predominantly from 3.7% in the pre-FAME period to 57.5% in the FAME period (*P* < .001). The pathogen identification rate also increased markedly during the FAME period, from 0.4% to 18.7% (*P* < .001; Table 2). Pathogens were identified in 36 of 193 cases (18.7%) during the FAME period: VZV most frequently (n = 24), followed by human parechovirus (n = 3); HSV-1, HHV-6, and *S pneumoniae* (n = 2 each); and enterovirus, *Listeria monocytogenes*, and group B *Streptococcus* (n = 1 each). Bacteria were detected by FAME in 2.1% of all cases (n = 4); in 3 of these, CSF culture was positive and antibiotics were administered. The remaining case had a positive blood culture for the pathogen (*S pneumoniae*) identified by FAME. In this case the CSF sample was obtained after the administration of antimicrobials. All 4 patients completed the course of antimicrobials for bacterial meningitis.

**Table 2.** Microbiological Examination of Cerebrospinal Fluid Samples During the Pre–FilmArray Meningitis/Encephalitis Panel (FAME) Period and FAME Period

Category	Pre-FAME Period	FAME Period	*P* Value
	Jan 2016 to Nov 2020 (n = 519)	Dec 2020 to Dec 2022 (n = 193)	
Overall testing			
No. of PCR tests performed	19 (3.7%)	111 (57.5%)	<.001
No. of positive PCR tests	2 (0.4%)	36 (18.7%)	<.001
Bacterial testing			
CSF culture performed	367 (70.7%)	139 (72.0%)	.732
Positive CSF culture	7 (1.3%)	3 (1.6%)	.836
Viral and bacterial PCR testing (positive)			
Herpes simplex virus (outsourcing)	0	NA	
Varicella zoster virus (outsourcing)	2 (0.4%)	NA	
Parvovirus (outsourcing)	0	NA	
Human herpesvirus 6 (outsourcing)	0	NA	
Enterovirus (outsourcing)	0	NA	
FAME			
Cytomegalovirus	NA	0	
Enterovirus	NA	1 (0.5%)	
Herpes simplex virus 1	NA	2 (1.0%)	
Herpes simplex virus 2	NA	0	
Human herpesvirus 6	NA	2 (1.0%)	
Human parechovirus	NA	3 (1.6%)	
Varicella zoster virus	NA	24 (12.4%)	
*Escherichia coli* K1	NA	0	
*Haemophilus influenzae*	NA	0	
*Listeria monocytogenes*	NA	1 (0.5%)	
*Streptococcus agalactiae*	NA	1 (0.5%)	
*Streptococcus pneumoniae*	NA	2 (1.0%)	
*Neisseria meningitidis*	NA	0	
*Cryptococcus neoformans*/*gattii*	NA	0	

Data are presented as No. (%) unless otherwise indicated.

Abbreviations: CSF, cerebrospinal fluid; FAME, FilmArray meningitis/encephalitis panel; NA, not applicable; PCR, polymerase chain reaction.

The results of the multivariable Poisson regression analyses are presented in Table 3. The LOS was longer in the FAME period compared with the pre-FAME period (adjusted rate ratio [aRR], 1.25 [95% confidence interval {CI}, 1.21–1.30]; *P* < .001). All antimicrobial DOT were shorter in the FAME period (aRR, 0.95 [95% CI, .90–1.00]; *P* = .034). While the antibacterial DOT was not statistically different in the FAME period compared to that of the pre-FAME period (aRR, 1.06 [95% CI, 1.00–1.13]; *P* = .063), the antiviral DOT was significantly shorter in the FAME period (aRR, 0.80 [95% CI, .71–.89]; *P* < .001).

**Table 3.** Multivariable Poisson Regression Analyses for Adjusted Rate Ratios of the FilmArray Meningitis/Encephalitis Panel (FAME) Period Compared With the Pre-FAME Period

Outcome	Adjusted Rate Ratio	(95% CI)	*P* Value
Length of stay	1.25	(1.21–1.30)	<.001
DOT for all antimicrobials	0.95	(.90–1.00)	.034
DOT for antibacterials	1.06	(1.00–1.13)	.063
DOT for antivirals	0.80	(.71–.89)	<.001

For each outcome, the multivariable Poisson regression analysis was conducted with dependent variables of period (the pre–FilmArray meningitis/encephalitis panel [FAME] period vs the FAME period), age group, sex, and seasonality. No offset term was set.

Abbreviations: CI, confidence interval; DOT, days of therapy.

## DISCUSSION

Our study showed a reduction in the total antimicrobial DOT and the antiviral DOT in the FAME period compared with the pre-FAME period. However, the LOS and antibacterial DOT did not decrease during the FAME period.

The reduction in total antimicrobial DOT in the FAME period was likely due to a reduction in antiviral use, given that antibacterial DOT was not statistically different between the 2 periods. A reduction in the use of acyclovir after the implementation of FAME was reported in a pediatric study in Japan [14]. Our study, which comprised an adult population, also confirmed this finding. Although some studies have reported cases of HSV meningitis/encephalitis, even when the first CSF sample was negative for the virus in a PCR test, our study suggests that a negative PCR result for HSV may help clinicians decide on whether to initiate or, if already on therapy, discontinue antivirals for patients with suspected HSV meningitis/encephalitis [16–19]. In our study, all cases with HSV meningitis/encephalitis had positive PCR results in the first CSF samples. However, for patients with a high suspicion of HSV infection, a negative PCR result for HSV needs to be interpreted cautiously, especially if the sample was taken in the early phase of the clinical course of the disease [20]. In addition, false-negative results of FAME have been reported in other pathogens. A systematic review showed that the sensitivity of detecting each bacterial pathogen by FAME was as low as 64.9% in *H influenzae*, 71.5% in *S agalactiae*, 70.9% in *E coli*, and 70.4% in *L monocytogenes* [21]. Thus, the negative FAME result does not necessarily rule out the possibility of bacterial meningitis by pathogens targeted in FAME. For patients with high pretest probability of bacterial meningitis caused by these pathogens, clinicians may cautiously need to evaluate the possibility of these pathogens even if the results of the FAME have been negative. On the other hand, the specificity of FAME has been reported to be high in general [21]. The positive bacterial result suggests that the pathogen causes the central nervous system infection.

The lack of a reduction in antibacterial use may be attributed to the difficulty of ruling out the possibility of bacterial meningitis in FAME-negative cases. While the FAME test has very high sensitivity and specificity for the pathogens included in the panel, it cannot detect other pathogens (eg, gram-negative bacteria other than *E coli* K1).

The study results showed an increased LOS in the FAME period compared to that in the pre-FAME period. A potential reason for the increased LOS was that the FAME test may have contributed to reduced hospitalization of patients. In other words, patients who would have stayed in the hospital for a short period without any rapid PCR test may have been discharged without hospitalization because of the reassuring results of the FAME test. Another possible explanation for the increased LOS in the FAME period is that patients in the FAME period were more severely ill at presentation than patients in the pre-FAME period. While the intensive care unit (ICU) admission rate in the FAME period demonstrated a higher trend compared with that in the pre-FAME period, this was not found to be statistically significant as assessed by the χ^2^ test. We did not include the disease severity factor (eg, ICU admission) as a dependent variable in the multivariable Poisson regression analyses because the implementation of the FAME test may have impacted disease severity through the results guiding patient management, including antimicrobial treatment.

Notably, the FAME period was during the coronavirus disease 2019 (COVID-19) pandemic when the incidence of many types of viral infections had significantly decreased. This may have affected the etiology of meningitis and encephalitis in both the pre-FAME and FAME periods. As many patients with viral meningitis, except for some cases (eg, HSV encephalitis), may have improved without antibacterials, fewer cases of viral meningitis in the FAME period may have been associated with an overall longer LOS and lack of reduction in antibacterial DOT in the FAME period. Further studies are required to evaluate the impact of the epidemiology of meningitis and encephalitis during and after the COVID-19 pandemic.

This study had some limitations. First, given the study design (retrospective cohort study), we could not fully eliminate the possibility of confounding factors even though we adjusted for some factors in the multivariable regression analysis. Second, the impact of the FAME panel on specific populations (eg, patients undergoing neurosurgery or those who are immunocompromised) could not be evaluated because these populations consisted of a very small proportion of participants in our study. Given that the causal pathogens of meningitis and encephalitis in these specific populations differ from those in the general population, our study results are not applicable to these specific populations. Third, our study excluded patients without pleocytosis, except for those aged <2 months. Some studies have reported that pathogens were often identified from the CSF in cases of meningitis or encephalitis without pleocytosis [22, 23]. The impact of FAME panels for those without pleocytosis should be evaluated in further studies.

In conclusion, this study revealed a significant reduction in antiviral use during the FAME period, whereas there was no decrease in the LOS and antibacterial use. Given the possibility of factors (eg, the COVID-19 pandemic) affecting the epidemiology of meningitis and encephalitis, the indications and impact of the FAME test should be evaluated with continuous monitoring of the epidemiology of meningitis and encephalitis and its clinical impact.

## Notes

***Author contributions.*** D. K. and T. Ki. conceptualized the study design and objectives. D. K., H. Y., S. Y., M. K., K. M., and F. N. supervised data collection. D. K., Y. U., T. A., H. N., R. S., M. O., T. Ka., N. S., K. T., M. S., and S. S. collected the data. D. K., T. Ki., and H. Y. accessed and verified laboratory results. D. K., M. S., and S. S. accessed and verified hospital admission data. T. Ki. did the statistical analyses. D. K. and T. Ki. wrote the first draft of the manuscript. D. K., T. Ki., Y. S., A. N., and R. N. did a literature search. All authors had full access to all data in this study, reviewed and approved the final manuscript, and had final responsibility for the decision to submit for publication.
